# FOXO3 upregulates and activates GSDME to trigger myeloma cell pyroptosis

**DOI:** 10.7150/ijbs.124782

**Published:** 2026-01-15

**Authors:** Yaner Wang, Yali Wang, Yaoli Cui, Yuanming He, Ye Yang, Wen Zhou, Longlong Liu, Hua Wang, Mo Liu, Yongqiang Wei, Zhenqian Huang, Xiaolei Wei, Xinliang Mao

**Affiliations:** 1 Department of Hematology, The Key Laboratory of Advanced Interdisciplinary Studies, The First Affiliated Hospital of Guangzhou Medical University; Guangdong Provincial Key Laboratory of Protein Modification and Degradation, School of Basic Medical Sciences, Guangzhou Medical University, Guangzhou 511436, China.; 2 Institute of Clinical Pharmacology, Science and Technology Innovation Center, Guangzhou University of Chinese Medicine, Guangzhou, 510405, China.; 3 Department of Clinical Pharmacology, College of Pharmaceutical Sciences, Guangzhou Medical University, Guangzhou 511436, P. R. China.; 4 School of Medicine & Holistic Integrative Medicine, Nanjing University of Traditional Medicine, Nanjing 210023, China.; 5 Cancer Institute, Central South University, Changsha, 410078, China.; 6 State Key Laboratory of Oncology in South China, Guangdong Provincial Clinical Research Center for Cancer, Sun Yat-sen University Cancer Center, Guangzhou, 510060, China.; 7 Sino-French Hoffmann Institute, Guangzhou Medical University, Guangzhou 511436, China.; 8 Department of Hematology, Southern Medical University, Guangzhou, 510515, China.

**Keywords:** myeloma, pyroptosis, FOXO3, GSDME, corylin

## Abstract

Induction of pyroptosis is considered as a novel strategy for the treatment of multiple myeloma, but the potential targets remain unknown. In the present study, we found that GSDME, a key executor of pyroptosis, is the mostly downregulated pyroptosis-related gene in MM cells and its low expression predicts poor prognosis of MM patients. Out of expectation, GSDME transcription is not markedly affected by epigenetic manners in MM cells. In contrast, GSDME expression is controlled by the transcription factor FOXO3. FOXO3 binds to the two recognition sites and upregulates GSDME. Moreover, FOXO3 specifically upregulates the BNIPL family proteins and activates Caspase-3 and GSDME therefore triggering MM cell pyroptosis. In addition, similar to GSDME, FOXO3 is also downregulated in MM and its restoration suppresses myeloma tumor growth. Furthermore, we found corylin, a flavonoid derived from Psoralea Fructus, activates the transcription of both FOXO3 and GSDME. As expected, corylin displays potent anti-MM activity in association with pyroptosis by upregulating FOXO3 and GSDME. In conclusion, FOXO3 is a novel transcription factor of GSDME. Restoration/activation of the FOXO3/GSDME axis could be a promising novel strategy for the treatment of MM.

## Introduction

Multiple myeloma (MM) is a not-yet curable hematological cancer derived from plasma cells. The current standard of care for MM largely depends on apoptosis induced by several classes of modalities, including glucocorticoids, chemotherapeutics, proteasome inhibitors, immunomodulators, targeted therapeutics, antibody drugs, and cell-based therapies including chimeric antigen receptor-T cells 1. However, even with the advanced comprehensive treatments, the 5-year survival rate is just higher than 50% 1, therefore, it is urgent to develop novel therapeutic modalities. In the last decade, several novel forms of cell death different from apoptosis have been well established towards cancer therapy^2^.

Pyroptosis is an emerging form of programmed cell death that has been originally associated with inflammasomes in response to virus or bacterial infection 3. Earlier studies focused on inflammatory cells that using gasdermin D (GSDMD) as the executor of pyroptosis. Upon activation, the N-terminal active form of GSDMD translocalizes to plasma membrane form a multiple-subunit pore resulting leakage of mature IL-18 and IL-1β 4. In 2017, GSDME, another member of the gasdermin family, was reported to provoke pyroptosis in cancer cells but in a caspase-3- dependent manner 5. Induction of pyroptosis not only induces cancer cell death but also overcomes chemoresistance and enhances anti-tumor immunity 6. Pyroptosis also plays a critical role in MM treatment. A panel of pyroptosis-related genes has been well established in predicting the prognosis of MM patients 7. Our recent study showed that proteasome inhibitors including bortezomib, induce MM cell pyroptosis in a GSDME-dependent manner, and these effects can be enhanced by the Bcl-2 inhibitor venetoclax^8^. Moreover, the natural product acevaltrate can overcome bortezomib resistance by inducing MM cell pyroptosis 9. Recent advancements have shown that induction of pyroptosis is a promising strategy in MM treatment. However, major targets in pyroptosis for MM treatment are not yet known.

In the present study, we identify GSDME is downregulated and it is modulated by FOXO3 in MM cells. Restoration of FOXO3 induces MM cell pyroptosis by promoting GSDME expression and activation in a caspase-3-dependent manner. We also found corylin, a natural product derived from a flavonoid *Psoralea Fructus*, promotes the transcription of FOXO3 and GSDME therefore exerting striking anti-MM activity.

## Methods

### Data acquisition

The expression profiles of pyroptosis-related genes (PRGs) and corresponding clinical information of 842 MM patients from the TCGA-MMRF CoMMpass dataset were obtained (https://portal.gdc.cancer.gov/repository) 10. To ensure data integrity and comparability, the gene expression data were subjected to normalization using the "limma" package, employing the scale method 11. The Genotype-Tissue Expression (GTEx) database was accessed to examine mRNA expression of the PRGs in the bone marrow of 70 healthy individuals.

### GEO dataset analyses

A DNA microarray for gene expression profile generated from healthy donors (n = 22) and MM patients with monoclonal gammopathy of undetermined significance (MGUS, n = 44), smoldering MM (SMM, n = 12) (GSE5900) 12 were analyzed for GSDME.

### Cells and cell culture

OCI-My5, OPM2 and RPMI-8226 were kindly provided by Dr. Keith A. Stewart, University of Toronto, Canada. All cells were maintained as described previously 8. Primary MM cells obtained from patients newly diagnosed with MM at the First Affiliated Hospital of Guangzhou Medical University. Informed written consent for research purposes was obtained from the patients. The study protocol was approved by the Review Board and Ethics Committee of the First Affiliated Hospital of Guangzhou Medical University (Approval No. ES-2024-201-01). All cells were cultured in IMDM medium supplemented with 10% fetal bovine serum (#F103, Vazyme, Nanjing, China).

### Reagents and antibodies

Corylin (Cat. #HY-N0236) and nigericin (Cat. #HY-100381) were provided by MedChem Express (Shanghai, China). The Natural Products Library was obtained from TargetMol (Wellesley Hills, MA). Etoposide (ETO, Cat. #S1225), doxorubicin (ADM, Cat. #E2516), and Z-DEVD-FMK (Cat. #S7312) were obtained from Selleck Chemicals (Houston, Texas, United State). Lipopolysaccharide (LPS, Cat. #L4391) was provided by Sigma-Aldrich (Shanghai, China). Doxycycline hyclate (DOX, Cat. #ST039A) was purchased from Beyotime (Shanghai, China). Antibodies against FOXO3 (Cat. #10849-1-AP), BAX (Cat. #50599-2-lg), BCL2 (Cat. #26593-1-AP), BCL2L1 (Bcl-xL, Cat. #10783-1-AP), MCL1 (Cat. #16225-1-AP) and BNIPL (Cat. #13262-1-AP) were obtained from Proteintech Group (Wuhan, China). Anti-caspase-3 (Cat. #9662S), Cytochrome c (Cat. #11940), VDAC1 (Cat. #4866), β-actin (Cat. #4967) and GAPDH (Cat. #2118) were obtained from Cell Signaling Technology, Inc. (Danvers, MA). Anti-GSDME (Cat. #ab215191) was obtained from Abcam (Shanghai, China). Anti-GSDMD (Cat. #NBP2-33422) was from Novus Biologicals (Centennial, Colorado).

### Plasmids

The pWPI-GSDME and human pWPI-GSDME-D270A were maintained in the lab 8. The full-length FOXO3 gene was amplified from HeLa cells by PCR with specific primers as shown in Supplemental Table S1. To generate a Tet-on expression system, the FOXO3 cDNA was inserted between Eco RI and Bam HI restriction sites of the pLV2-TRE3GS-3×Flag-TetOne-Puro lentiviral vector. The pLV3-BNIPL plasmid was purchased from MiaolingBio (Wuhan, China). The lentiviral shRNAs against FOXO3 (shFOXO3) and the negative control (shNC) were purchased from IGE Biotechnology LTD (Guangzhou, China). The lentiviral shRNAs against BNIPL (shBNIPL), Bcl-xL (shBcl-xL) and the negative control (shNC) were purchased from Beijing Tsingke Biotech Co., Ltd. (Guangzhou, China). Primers were shown in Supplemental Table S2.

### Construction of the truncated GSDME regulatory regions and mutants

Genomic DNA was extracted from HEK293T cells according to the manufacturer's protocol (Beyotime, Shanghai, China). To generate the truncates regulatory region of GSDME, the site-directed mutagenesis PCR was performed as shown previously 13. The specific primers were shown in Supplemental Table S3.

### Lactate dehydrogenase (LDH) release assay

The concentration of LDH released into cell culture media was determined by LDH Cytotoxicity Assay Kit (Beyotime) as reported previously 8.

### Transcriptomic sequencing

Cells were collected, resuspended in TRIzol, and submitted to IGE Biotechnology for transcriptome sequencing. RNA integrity was assessed using the Agilent 2100 system, followed by library preparation and quality control. Libraries were then pooled based on effective concentration and target data output for Illumina sequencing.

### Dual-luciferase reporter assays

The constructs of GSDME regulatory sequences or mutants, along with the internal control vector Renilla, were co-transfected into HEK293 cells. Luciferase assays were performed after 24h using the Dual Luciferase Reporter Assay System (Promega, Beijing, China) as described previously 13.

### Chromosomal immunoprecipitation (ChIP)

The ChIP assay was performed using the ChIP kit (Beyotime) as described previously 13. The fragment of the -212~-300 regulatory region of GSDME from treated cells was amplified by PCR using the primers: forward 5'-GAAGTGGGTCACAATAAA-3' and reverse 5'-CATAAGACAATACGAGGG-3'.

### Reverse transcription-polymerase chain reaction (RT-PCR) and quantitative real-time reverse transcriptase-polymerase chain reaction (qRT-PCR)

RNA extraction was performed using the RNAse Plus Animal RNA Isolation Kit with Spin Column (Beyotime) as instructed by the manufacturer. The cDNA synthesis from the extracted RNA was using the cDNA Synthesis Mix (EasyScript First-Strand cDNA Synthesis SuperMix (TransGen Biotech, Beijing, China) as described previously 8. For endpoint verification, load PCR products onto agarose (Sangon Biotech, Shanghai, China) gel prepared in 1× TAE/TBE with ethidium bromide/SAFE dye (RT-PCR). The specific primers were listed in Supplementary Table S4. qRT-PCR was conducted using TB Green Premix Ex Taq II (Tli RNaseH Plus) with ROX (Takara, Dalian, China). The mRNA expression levels were normalized using the reference gene GAPDH, and the specific mRNA expression levels were quantified using the 2^-∆∆CT^ method. The specific primers were listed in Supplementary Table S5.

### Measurement of IL-18 and IL-1β by Enzyme-Linked ImmunoSorbent Assay (ELISA)

MM cells were treated with Nigericin (5 μM) and LPS (500 ng/mL) for 6 h or were infected with FOXO3-overexpression lentivirus for 96 h. Cell supernatants were collected and centrifuged at 300 g for 10 minutes to remove debris. IL-18 and IL-1β levels were quantified using ELISA kits (Jianglai Biotechnology Co., Ltd, China) according to the manufacturer's protocol. Optical density was measured at 450 nm using a microplate reader, and cytokine concentrations were calculated based on standard curves. Experiments were performed in triplicates, and data were normalized to untreated controls.

### High throughput screen

HEK293T cells expressing GSDME promoter-driving luciferase was incubated with individual compounds from a natural product library L6000 (TargetMol, Wellesley Hills, MA) for 24 h, followed by luciferase determination as described previously 14. Compounds that raised luciferase activity > 2 folds were subjected to further studies.

### MM xenografts

Two types of MM xenograft models were established in the study. The first one was derived from MM cells containing a stably expressing Tet-on inducible FOXO3 system in female immunodeficient BALB/c nude mice. When tumors were palpable, mice were randomized into two groups, mice were administrated with 3% sucrose solution containing Doxycyline (DOX) (3.2 mg mL^-1^) or not 15. The drink was refreshed every other day throughout the experiment. The second one was established by inoculating OCI-My5 and OPM2 cells into five-week-old female immunodeficient BALB/c nude mice (1×10^7^ cells/mice). When tumors were palpable, mice were randomly divided into two groups, each was received corylin (50 mg kg^-1^, *i.p.*, three times a week) or vehicle only. All mice were obtained from the Vital River Laboratory Animal Technology Co., Ltd. (Beijing, China). These experiments were approved by the Review Board for Animal Welfare and Ethics of Guangzhou Medical University (Approval No. GY2024-722).

### Statistical analysis

To assess the statistical differences between control and experimental groups, Student's t-test was performed using the GraphPad Prism 9.0 software. The statistical significance was considered as a *p*-value less than 0.05 if not otherwise specified.

## Results

### GSDME is downregulated in MM and its low expression is correlated with poor outcome of MM patients

**Figure 1:**
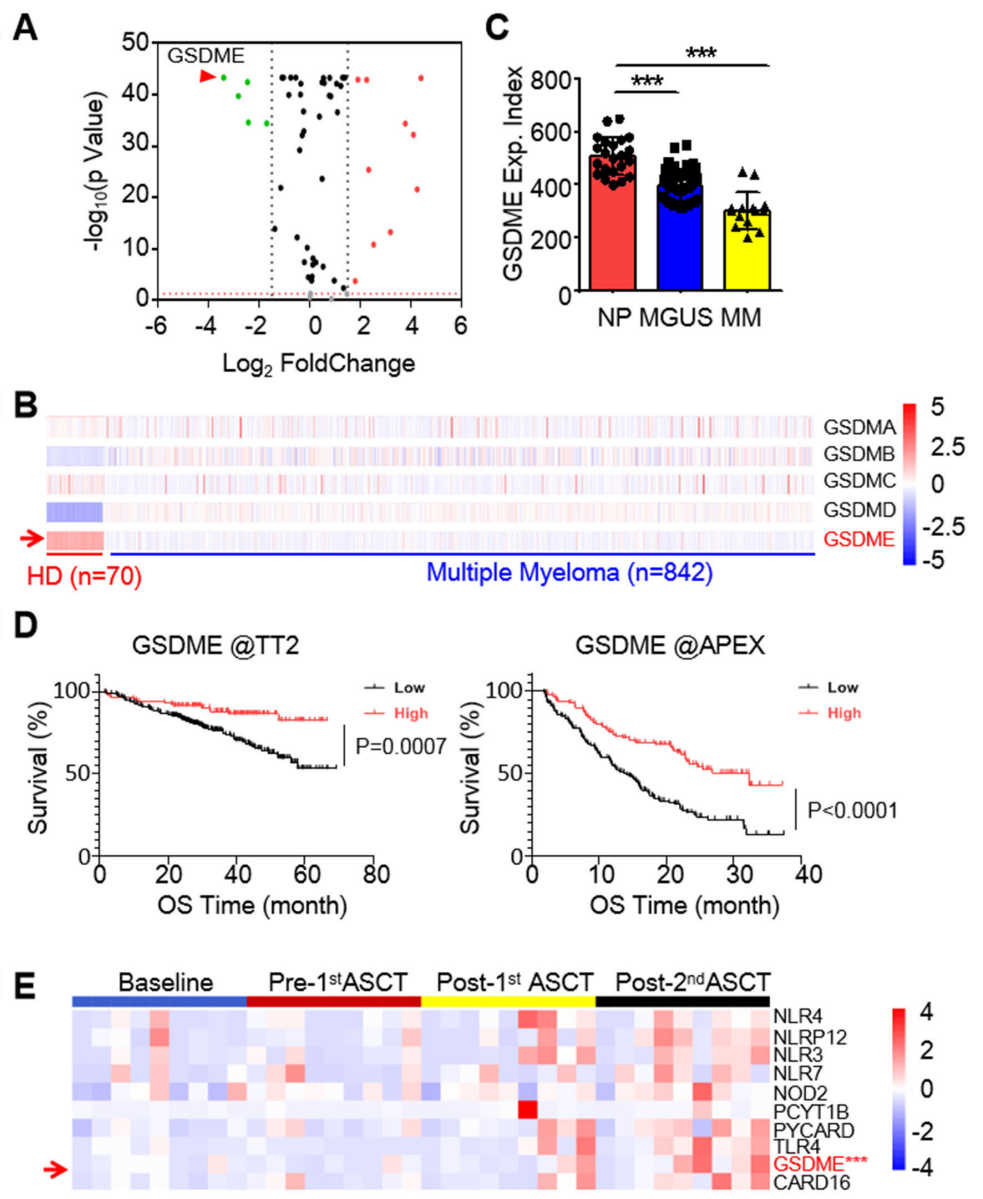
** GSDME is downregulated in myeloma and predicts poor prognosis.** A, The expression profile of 57 pyroptosis-related genes in MM patients against healthy volunteers. Data were presented as a volcano plot. B, The expression profile of gasdermins in individual bone marrow cells from healthy donors and MM patients. C, GSDME expression was analyzed by qPCR from normal plasma cells (NP), MGUS cells and MM cells. D, The association of overall survival and the expression of GSDME from the TT2 and APEX datasets. E, The expression profiles of GSDME and several other pyroptosis-related genes in MM patients at the baseline, pre- and post- autologous stem cell transplantation (ASCT). ***, *P*< 0.001.

To identify potential anti-MM targets among the known pyroptosis-related genes (PRGs), we analyzed transcriptomic profiles of 842 MM patients from the TCGA-MMRF CoMMpass dataset 10 and 70 healthy donors (HD) from the Genotype-Tissue Expression (GTEx) database. It revealed that several genes were markedly downregulated in MM cells, and GSDME was the most downregulated one (Fig. 1A). Notably, among the five known pyroptosis executors, GSDME was the only one strikingly downregulated (Fig. 1B). Moreover, GSDME was progressively downregulated throughout the myelomagenesis process (Fig. 1C). The expression level of GSDME was positively associated with overall survival rate of MM patients based on two well-known datasets - Total Therapy 2(TT2) 16 and Assessment of Proteasome Inhibition for Extending Remissions (APEX) 17 (Fig. 1D). To confirm this finding, we also measured GSDME expression in bone marrow cells from MM patients before and after autologous stem cell transplantation (ASCT). Our analysis showed that GSDME expression increased after ASCT, especially following the 2nd ASCT (Fig. 1E). These results indicate that GSDME is downregulated in MM patients, while its high expression is associated with a favorable prognosis.

### GSDME expression is modulated by FOXO3 in MM cells

**Figure 2:**
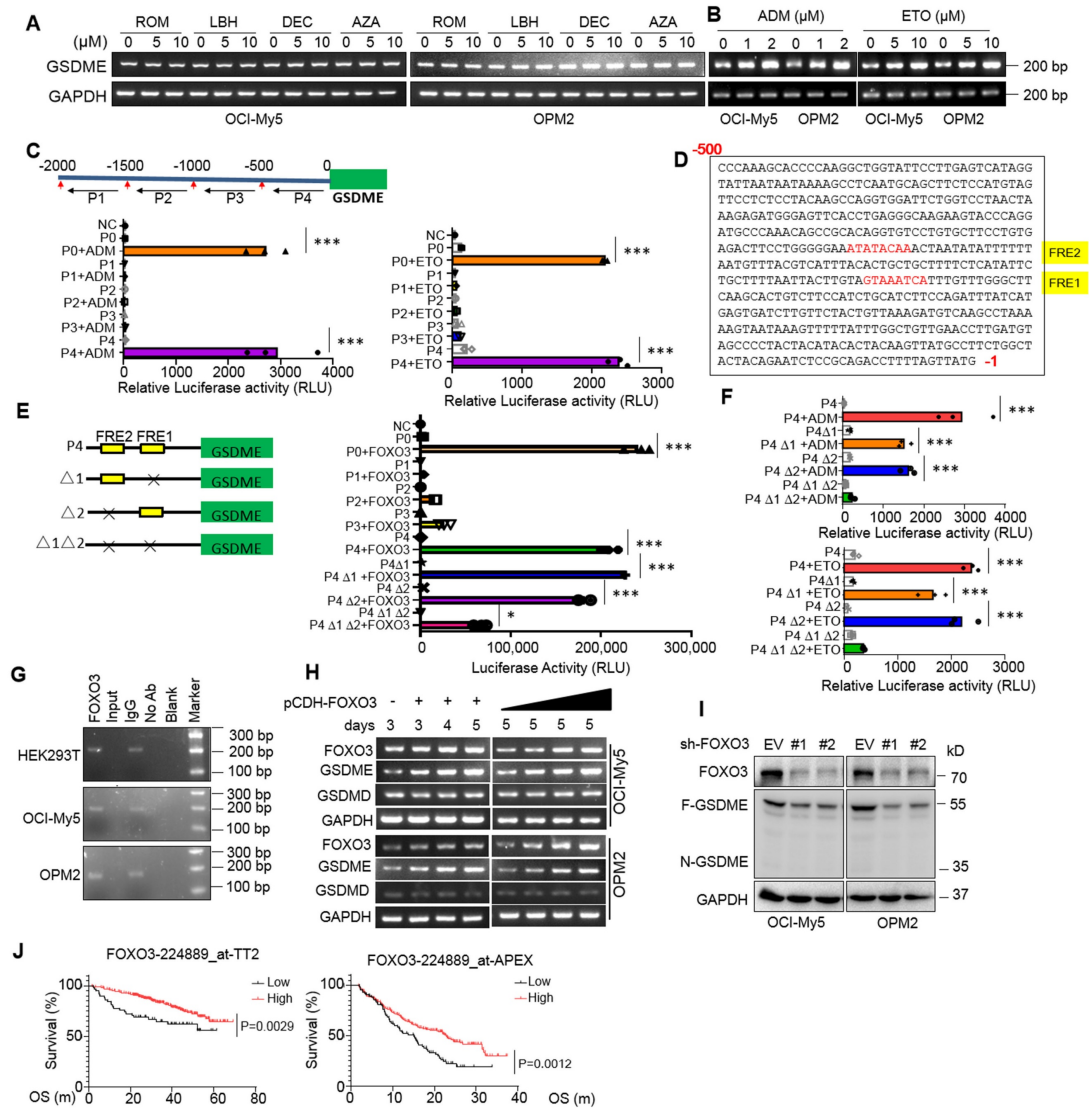
** GSDME is upregulated by the transcription factor FOXO3 in myeloma cells.** A-B, GSDME was measured by RT-PCR after MM cells were treated with epigenetic (A) or non-epigenetic (B) drugs. ROM, Romidepsin; LBH, LBH589; AZA, 5'-Azacytidine; DEC, decitabine. ADM, doxorubicin; ETO, etoposide. C, The fragments of the upstream 2000-nt promoter region of GSDME were cloned into the pGL3 luciferase vector, respectively, followed by ADM or ETO treatment. The luciferase activity was measured after 24 h. D, The FOXO3 recognition sites were identified in the promoter region (-500 ~-1nt) of GSDME. E, The fragments of GSDME regulatory region lacking the potential FOXO3 recognition elements (FREs) were inserted into a pGL3 vector, followed by co-transfection with FOXO3 into HEK293T cells. The luciferase activity was measured after 24 h. F, HEK293T cells expressing luciferase plasmids from E were treated with ADM or ETO for 24 h, followed by measurement of luciferase activity. G, The ChIP assays were performed to determine the binding between FOXO3 and its recognition elements (FRE) in the GSDME regulatory region. The FRE precipitated with a FOXO3 specific antibody was analyzed by RT-PCR. H, OCI-My5 and OPM2 cells were infected with lentiviral FOXO3 as indicated, followed by RT-PCR to measure GSDME expression. I, MM cells were knocked down BNIPL by its specific shRNA for 96 h, followed by WB assays to evaluate GSDME expression.J, The association between the expression of FOXO3 and overall survival rates of MM patients were analyzed in two different datasets TT2 and APEX. OS, overall survival; m: month. *, *P*<0.05**; *P*<0.01; ***, *P*< 0.001.

Previous reports showed GSDME expression might be suppressed by hypermethylation in some solid tumors and induction of GSDME by decitabine (DEC) could lead to breast cancer cell pyroptosis 5, 18. To find out whether GSDME downregulation in MM cells was modulated by common epigenetic mechanisms: DNA methylation and histone acetylation, MM cells were treated with DEC and 5'-aza-cysticidine (AZA), two inhibitors of DNA methyltransferase, or panobinostat (LBH589) and romidepsin (ROM), two typical inhibitors of histone deacetylases for MM treatment, but neither drugs induced GSDME expression (Fig. 2A); however, adriamycin (ADM) and etoposide (ETO), both are topoisomerase inhibitors, strikingly upregulated GSDME (Fig. 2B). These results suggest that GSDME in MM cells is not suppressed by methylation or acetylation.

It is known that gene transcription is finely tuned by its specific transcription factors (TFs) that recognize and bind to the specific elements at the gene regulatory region 13. To find out whether GSDME expression promoted by ADM and ETO in MM cells was controlled by a specific TF, we cloned the upstream 2000 nucleotides (nt) of the transcription start site (TSS), and this 2000-nt fragment was further divided into four segments as shown in Fig. 2C and each was cloned into a luciferase reporter vector, respectively. As shown in Fig. 2C, both ADM and ETO promoted the luciferase expression driven by both the full-length 2000-nt (P0) and the -500~-1-nt fragment (P4) but not by others, suggesting that ADM and ETO promoted GSDME expression probably in association with the specific TFs located at P4. We further analyzed the potential TFs of GSDME via JASPAR, a database of transcription factor binding profiles (https://jaspar.elixir.no) and found two potential FOXO3 recognition elements (FREs) in the P4 but not in other fragments (Fig. 2D). Therefore, we evaluated the effects of FOXO3 on the luciferase reporter by deleting FRE1 and/or FRE2. As shown in Fig. 2E, FOXO3 activated the full-length (P0) and P4-driving luciferase expression but showed no activity on P1, P2 or P3-driving ones. Moreover, when one of the two potential FREs was deleted, the expression of luciferase was not altered by FOXO3; in contrast, when both were deleted, FOXO3 failed to trigger luciferase expression (Fig. 2E). This finding was also confirmed in assays by ADM and ETO treatment (Fig. 2F). Furthermore, FOXO3 could bind to the regulatory region of GSDME as assayed by ChIP (Fig. 2G) and ectopic FOXO3 upregulated GSDME but not GSDMD expression in MM cells in a concentration- and time-dependent manner (Fig. 2H). Consistent with these findings, knockdown of FOXO3 downregulated the expression of GSDME (Fig. 2I). Lastly, FOXO3 was downregulated in primary MM cells and its high expression predicted a favorable clinical prognosis (Fig. 2J). Therefore, we found that FOXO3 binds to GSDME and promotes its transcription.

### FOXO3 induces myeloma cell pyroptosis by activating caspase-3 and GSDME

**Figure 3:**
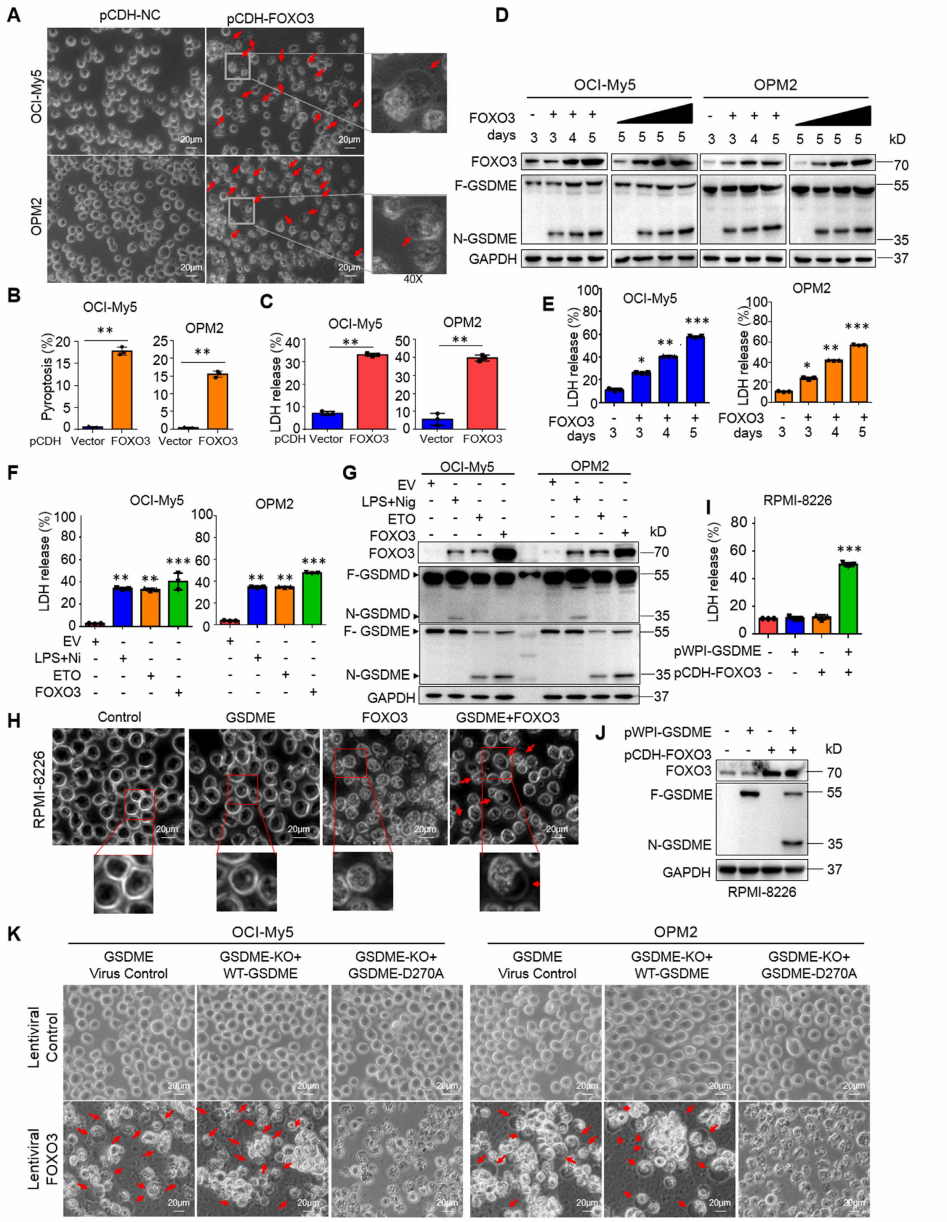
** FOXO3 induces myeloma cell pyroptosis by activating GSDME**. A-C, FOXO3 was introduced into MM cell lines via lentivirus for 96 h. Cell pyroptosis was evaluated by using phase-contrast microscopy(A), statistical analysis on pyroptotic cells (B) and LDH release in culture media (C). D-E, MM cells were infected with lentiviral FOXO3 by increased viral particles or infection time as indicated followed by IB (D) and LDH measurement (E). F-G, MM cells were infected with lentiviral FOXO3 for 96 h or treated with ETO (20 μg mL^-1^) or LPS (500 ng mL^-1^)/Nigericin (Nig, 5 μM) for 24 h, followed by measurement of LDH at the culture media (F) or IB against specific proteins as indicated (G). H-J, GSDME lacking RPMI-8226 cells were infected with lentiviral GSDME, FOXO3 alone or together for 96 h, followed by phase-contrast microscopy analysis (H), LDH measurement in culture media (I) and IB assays (J). K, OCI-My5 and OPM2 were knocked out (KO) GSDME, followed by infection with FOXO3 along with wild-type (WT) or D270A mutant GSDME. Cell pyroptosis was analyzed by phase-contrast microscope. *, *P*<0.05**; *P*<0.01; ***, *P*< 0.001. Scale bar = 20 μm. Arrows indicated pyroptotic cells.

Given FOXO3 can promote GSDME, a key executor of pyroptosis, we wondered whether FOXO3 could induce MM cell pyroptosis. To this end, we infected lentiviral FOXO3 into MM cells and found that ectopic FOXO3 strikingly induced MM cell pyroptosis featured with balloon-like appearance (Fig. 3A-B) and increased LDH release (Fig. 3C), a hallmark of pyroptosis 5, in a time- and concentration-dependent manner (Fig. 3D-E). Moreover, similar to ETO, FOXO3 activated GSDME but not GSDMD as that induced by LPS/Nig (Fig. 3F-G).

To find out whether GSDME is essential in FOXO3-induced MM cell pyroptosis, lentiviral GSDME was introduced into RPMI-8226, a MM cell line lacking GSDME but expressing GSDMD and GSDMC 5, 8, followed by introduction of FOXO3. The results showed that FOXO3 alone was not able to trigger RPMI-8226 cell pyroptosis (Fig. 3H). However, when GSDME was ectopically introduced, the cells underwent pyroptosis, as evidenced by characteristic balloon-like cell morphology (Fig. 3H), LDH release (Fig. 3I), and GSDME activation (Fig. 3J). Moreover, in OCI-My5 and OPM2 cells, GSDME knockout abolished FOXO3-induced pyroptosis, while re-expression of the wild-type (WT) GSDME but not the dead D270A mutant restored pyroptosis in the presence of FOXO3 (Fig. 3K, Supplemental Fig. S1A-B). Furthermore, incubation of MM cells with z-DEVD-FMK, a caspase-3-specific inhibitor, significantly reduced FOXO3-induced pyroptosis compared to the control (Supplemental Fig. S1C-D). Lastly, we examined the secretion of matured cytokines IL-18 and IL-1β from MM cells infected with FOXO3 and found that, compared with the treatment of LPS/Nig, the concentrations of both IL-18 and IL-1β were very low (Suppl. Fig. S1E-H), which was consistent with the previous findings that IL-18 and IL-1β prefer to be released via the GSDMD pores but not the GSDME pores given IL-18 and IL-1β are cleaved to be mature by Caspase-1, the same activator of GSDMD. These findings demonstrated that FOXO3-induced MM cell pyroptosis requires both caspase-3 and GSDME.

### FOXO3 induces BNIPL to activate caspase-3 and GSDME in MM cells

**Figure 4:**
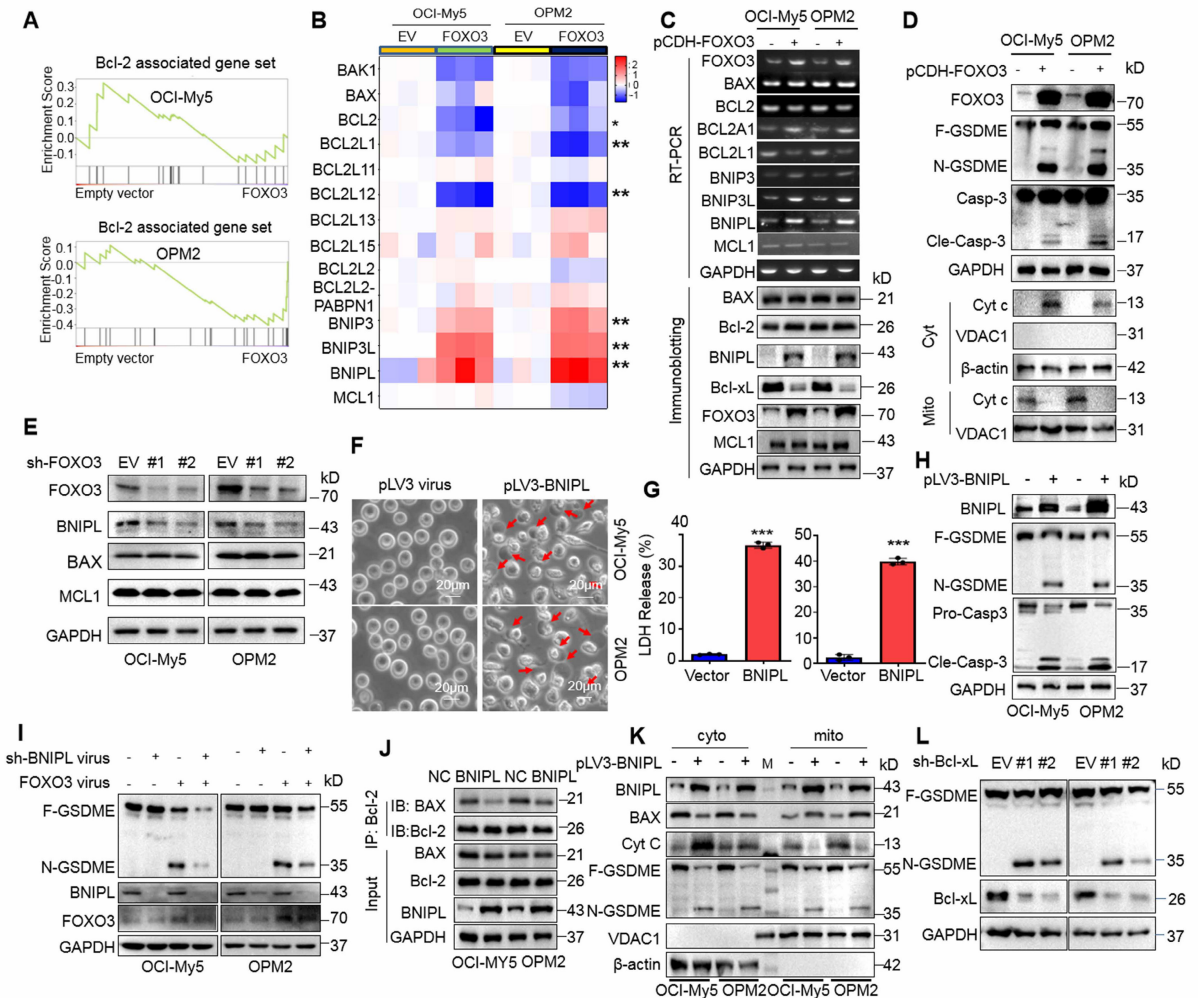
** FOXO3-induced BNIPL is critical for MM cell pyroptosis.** A-B, MM cells were infected with lentiviral FOXO3 for 96 h, followed by transcriptomic sequencing and the results were presented as GSEA plots (A) and heatmaps (B). C, The transcriptome sequencing findings were validated by RT-PCR and IB. D, MM cells infected with lentiviral FOXO3 were subjected to measurement of cyt c in the mitochondrial and cytosol fractions. E, MM cell were knocked down FOXO3, followed by IB assays to examine the expression of representative proteins. F-H, OCI-My5 and OPM2 cells were infected with lentiviral BNIPL for 96 h, followed by pyroptotic cell morphology analysis (F), LDH measurement (G) and GSDME measurement (H). I, MM cells were co-infected with BNIPL and FOX3 lentivirus for 96 h, followed by IB assays against specific proteins as indicated. J, MM cell lysates were collected for co-immunoprecipitation and IB assays following ectopic overexpression of BNIPL in OCI-My5 and OPM2 cells. K, MM cells were infected with BNIPL lentivirus for 96 h, followed by isolation of the cytosol and mitochondrial fractions and IB assays as indicated. L, MM cells were knocked down Bcl-xL by its specific shRNA for 96 h followed by IB assays. ***, *P*< 0.001. Arrows indicated pyroptotic cells.

To elucidate the specific mechanism, we performed transcriptomic sequencing on OCI-My5 and OPM2 infected with lentiviral vectors carrying FOXO3. Gene Set Enrichment Analysis (GSEA) revealed a panel of Bcl-2-associated genes was strikingly altered in both cell lines (Fig. 4A). Specifically, Bcl2L1 (also known as Bcl-xL) and BCL2L12 were markedly downregulated, while the BNIP family proteins including BNIPL, BNIP3 and BNIP3L were strikingly upregulated by FOXO3. Unexpectedly, the major players Bcl-2 and BAX were not significantly affected (Fig. 4B). These findings were further confirmed by both RT-PCR and IB assays (Fig. 4C). Moreover, cytochrome c (cyt c) was significantly increased in the cytosol but reduced in mitochondria, which was associated with the activation of caspase-3 and GSMDE (Fig. 4D). Consistently, when FOXO3 was knocked down, the downstream genes BNIPL was downregulated, but Mcl-1 and BAX remained unchanged (Fig. 4E), which was consistent with the results from FOXO3 overexpression. Given that Bcl-2 inhibitors have been reported to induce cancer cell pyroptosis^19^, we wondered whether the upregulation of BNIP family proteins was involved in MM cell pyroptosis. To this end, MM cells were infected with lentiviral BNIPL, a representative BNIP family protein. Our results demonstrated that ectopic expression of BNIPL dramatically induced MM cell pyroptosis (Fig. 4F) as evidenced by LDH release (Fig. 4G) and the activation of caspase-3 and GSDME (Fig. 4H). Consistently, knockdown of BNIPL partly ablated GSDME activation induced by FOXO3 (Fig. 4I), further suggesting that BNIPL plays a critical role in FOXO3-induced MM cell pyroptosis. Furthermore, BNIPL prevented the interaction between Bcl-2 and BAX (Fig. 4J) and BNIPL also promoted cyt c release from mitochondria, a key event in the initiation of pyroptosis (Fig. 4K). Lastly, given Bcl-xL was downregulated by FOXO3, we also examined the effects of shBcl-xL on pyroptosis and found that knockdown of Bcl-xL strikingly activated GSDME (Fig. 4L). Given Bcl-xL also prevents BAX from access to mitochondria and liberated BAX can penetrate the mitochondrial membrane and leads to MM cell pyroptosis 8, these findings collectively demonstrated that FOXO3 induces MM cell pyroptosis by modulating Bcl-2-associated genes, such as BNIPL and Bcl-xL, thereby leading to the activation of caspase-3 and GSDME.

### FOXO3 triggers ROS production and reduces mitochondrial membrane potential

The above studies showed that FOXO3 regulates the expression of the Bcl-2-associated genes and induces cyt c release from mitochondria, we therefore wondered whether FOXO3 impaired mitochondrial function. To this end, MM cells were infected with lentiviral FOXO3 or BNIPL, followed by ROS detection and TMRE staining. The results showed that both FOXO3 and BNIPL expression led to increased ROS production (Supplemental Fig. S2A and S3A) and decreased mitochondrial membrane potential (MMP) as indicated by TMRE staining (Supplemental Fig. S2B and S3B). To further find the role of ROS in FOXO3-induced cell pyroptosis, we established Tet-on/FOXO3/OPM2 and Tet-on/FOXO3/OCI-My5 cell lines. These cell lines were treated with N-acetyl cysteine (NAC) for 5 h before cells were treated with doxycycline (DOX) for 48 h. The results showed that DOX induced FOXO3 expression and GSDME activation, but in the presence of NAC, FOXO3-activated GSDME was strikingly reduced (Supplemental Fig. S2C), suggesting that ROS plays a critical role in FOXO3-induced MM cell pyroptosis. These findings are consistent with mitochondrial damage observed during pyroptosis, cyt c release, and caspase-3 activation 8. Therefore, FOXO3 induces MM cell pyroptosis with a strong association with mitochondrial damage.

### Induction of FOXO3 prevents the growth of MM xenografts via induction of pyroptosis

**Figure 5:**
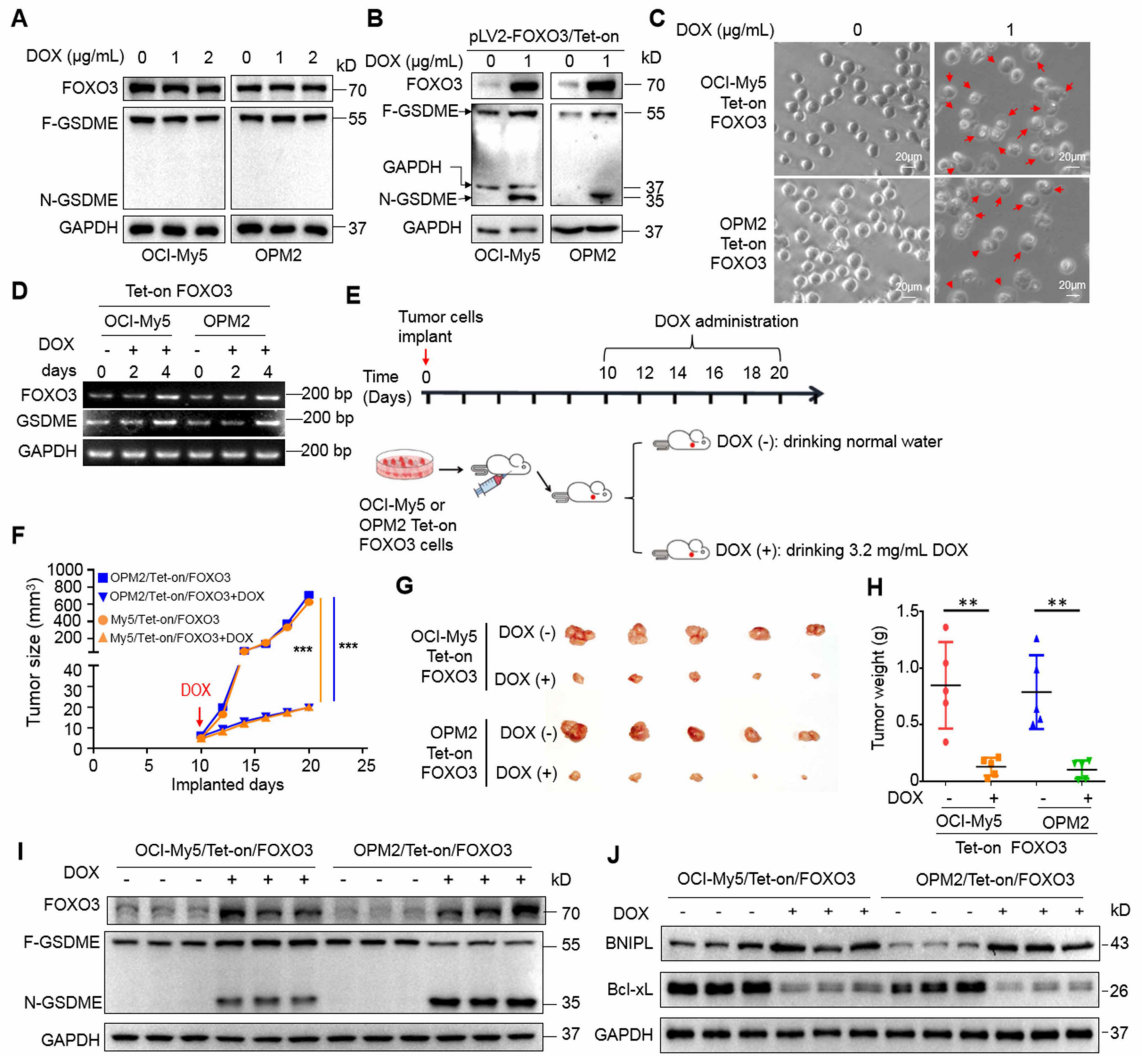
** Induction of FOXO3 leads to GSDME activation and arrests growth of myeloma xenografts in mice.** A, MM cells were treated with doxycycline (DOX) at indicated concentrations followed by IB assays. B-C, MM cells stably carrying a Tet-on-inducible FOXO3 system, followed by IB assays against FOXO3 and GSDME (B) and pyroptotic analysis by phase-contrast microscopy (C). D, MM cells carrying Tet-on/FOXO3 were treated with DOX as indicated, followed by RT-PCR for the transcription of FOXO3 and GSDME. E-H, OCI-My5 and OPM2 cells carrying the pLV2-FOXO3/Tet-on system were transplanted into immunodefficient mice for 10 days (d), followed DOX treatment for continued 12 days (E), measurement of tumor sizes (F-G), measurement of tumor weights (H). I, the expression of FOXO3 and GSDME in tumor tissues were analyzed by IB. J, the measurement of BNIPL and Bcl-xL in tumors treated with DOX or not. **, *P*< 0.01; ***, *P*< 0.001. Arrows indicated pyroptotic cells.

We next wondered whether induction of FOXO3 exhibits anti-MM activity *in vivo* in association with GSDME. To this end, a Tet-on FOXO3-inducible expression system was constructed and stably introduced into OPM2 and OCI-My5 cells. DOX treatment strikingly induced FOXO3 expression and GSDME activation as well as increased pyroptosis in the Tet-on cells, but not in the parental cells (Fig. 5A, B and C). Notably, DOX also promoted the transcription of both FOXO3 and GSDME (Fig. 5D). Subsequently, these Tet-on/FOXO3 cells were injected into nude mice to establish MM xenograft models. When tumors became palpable, mice were randomly divided into two groups, one was given the sucrose solution containing DOX and the other was given sucrose solution only for 12 days (Fig. 5E). Tumor sizes were monitored every other day and the results showed that DOX strikingly inhibited growth of xenografts carrying the Tet-on inducible FOXO3 plasmids (Fig. 5F). Both tumor sizes and weight were significantly reduced (Fig. 5G and H). Moreover, DOX strikingly induced FOXO3 expression and activated GSDME in tumor tissues (Fig. 5I) in a manner consistent with that in the vitro assays (Fig. 5B). BNIPL was also induced while Bcl-xL was downregulated by DOX in MM xenografts (Fig. 5J). Therefore, the induction of FOXO3 displays potent anti-MM activity *in vivo* by activating GSDME via modulating the Bcl-2-associated proteins.

### Corylin triggers MM cell pyroptosis and suppresses MM tumor growth *in vivo* by upregulating FOXO3 and activating GSDME

**Figure 6:**
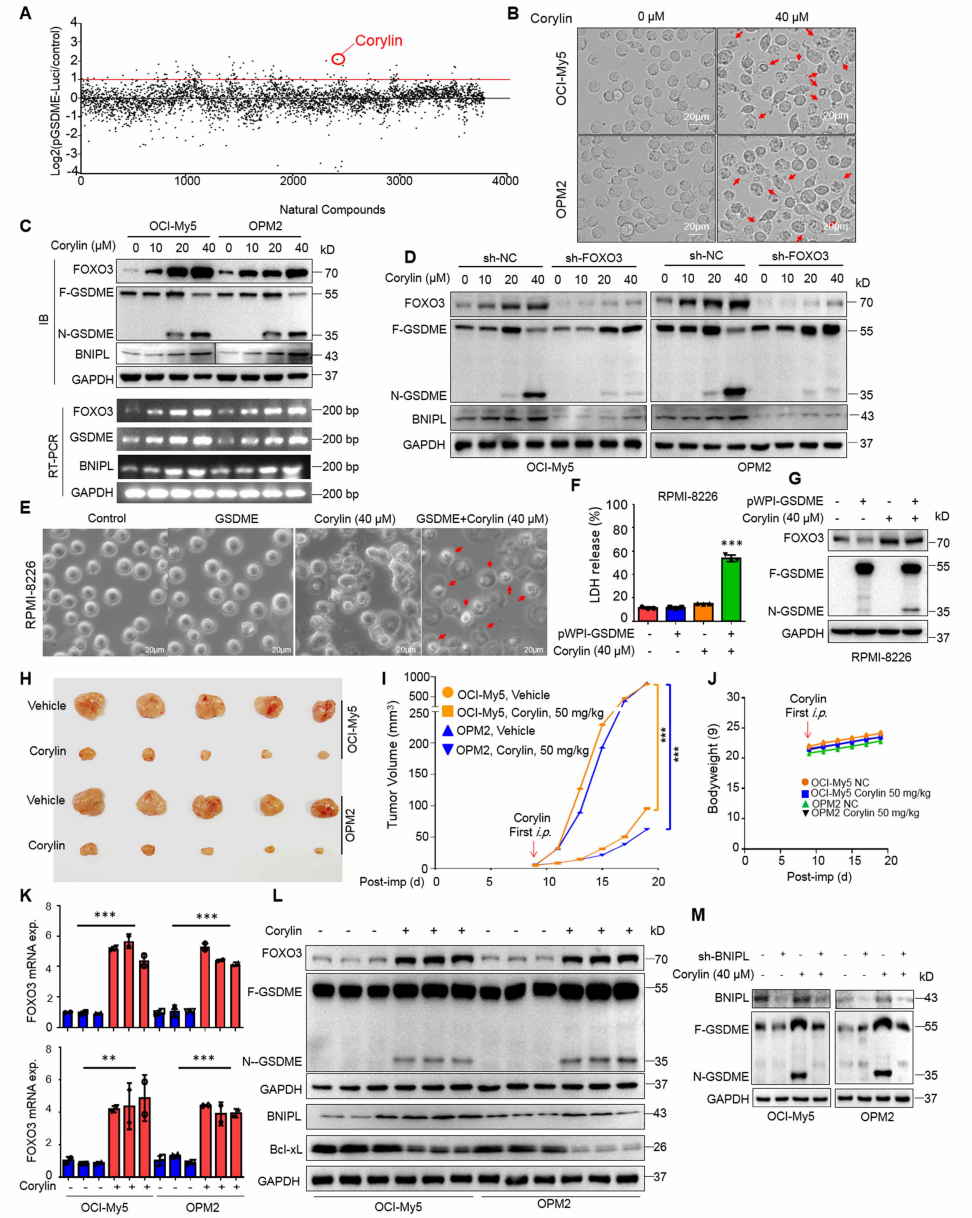
** Corylin induces myeloma cell pyroptosis by upregulating FOXO3 and GSDME.** A, HEK293T cells transfected with the pGSDME-P4.Luci construct were incubated for 24 h with each compound from the library composed of 3760 natural products (TargetMol) before being subjected to luciferase activity measurement. The Log2 (the ratio of luciferase activity against control) was calculated. B-C, MM cells were treated with corylin for 24 h, followed by phase-contrast microscope (B) and IB assays (C). D, FOXO3 was knocked down before MM cells were treated with corylin for 24 h. Cell lysates were prepared for IB assays against FOXO3 expression and GSDME activation. E-G, GSDME-lacking RPMI-8226 cells were infected with lentiviral GSDME, followed by corylin treatment. Pyroptotic cells were analyzed by phase-contrast microscope (E), LDH measurement (F) and IB assay against GSDME and FOXO3 (G). H-J, MM cells were implanted into the flank of immunodefficient nude mice. When tumors were palpable, mice were randomly divided into two groups, one was treated with vehicle, the other one was given corylin (50 mg/kg) for 12 days. Tumors were excised at the end of the experiment (H), analysis of tumor growth(I) and mice bodyweights (J). Post-imp, post implantation. K-L, Tumor tissues were subjected to measurement of FOXO3 and GSDME by qRT-PCR (K) and IB assays (L). M, OCI-My5 and OPM2 cells were infected with shBNIPL lentivirus for 96 h, followed by treatment with corylin (40 μM) for 24 h. Cell lysates were then prepared for IB assays. **, *P*< 0.01; ***, *P*< 0.001. Arrows indicated pyroptotic cells.

Given that the induction of FOXO3 and GSDME expression by DOX could induce MM cell pyroptosis, we wondered whether any natural products could also display the similar activity. To this end, we designed a luciferase-based drug screening system by cloning the FOXO3-recognition element-containing promoter of GSDME into a luciferase vector. Cells were then treated with individual compounds from a library containing 3760 natural products. After three rounds of screens, corylin, a natural product isolated from the Chinese medicinal herb *Psoralea corylifolia L. (Fabaceae)*, was identified for further studies (Fig. 6A). Corylin displayed potent activity against GSDME-expressing OCI-My5 and OPM2 cells but was less effective against RPMI-8226 cells that lack GSDME 8 (Fig. 6B and Supplemental Fig. S4A). As expected, corylin upregulated the expression of FOXO3, GSDME, and BNIPL at both RNA and protein levels (Fig. 6C). When FOXO3 was knocked down, BNIPL expression and GSDME activation induced by corylin were almost completely abolished (Fig. 6D). Lastly, we found that corylin failed to induce pyroptosis in RPMI-8226 but in OPM2 and OCI-My5 cells (Fig. 6E and Supplemental Fig. S4B, C and D), suggesting corylin-triggered MM cell pyroptosis was GSDME-dependent. To confirm this hypothesis, GSDME was ectopically expressed in RPMI-8226 cells and it showed these cells underwent pyroptosis induced by corylin as evidenced by morphology changes (Fig. 6E), LDH release (Fig. 6F and Supplemental Fig. S4D) and GSDME activation (Fig. 6G).

Given ectopic expression of FOXO3 impairs mitochondrial function during pyroptosis, we next evaluated corylin on mitochondria. It showed that corylin also impaired mitochondrial function, as evidenced by increased ROS production and decreased MMP (Supplemental Fig. S5A and B). Moreover, we also found that knockdown of BNIPL almost abolished MM cell pyroptosis triggered by corylin as assayed by morphological alteration and LDH release, two hallmarks of pyroptosis (Supplemental Fig. 6A, B and C). Therefore, corylin triggers MM cell pyroptosis by modulating the expression of BNIPL and impairing mitochondrial function as that induced by FOXO3.

Next, we evaluated the activity of corylin against MM *in vivo*. MM xenograft models derived from OCI-My5 and OPM2 cells were established in immunodeficient nude mice. When the tumors became palpable, mice were treated with corylin or vehicle for 12 days. The results showed that corylin almost suppressed MM tumor growth in both models (Fig. 6H and I) and caused no overt toxicity in terms of bodyweight (Fig. 6J). Moreover, corylin markedly promoted the expression of both FOXO3 and GSDME (Fig. 6K) and activated GSDME in tumors (Fig. 6L). Consistent with the *in vitro* study, corylin also downregulated Bcl-xL but upregulated BNIPL expression in tumors (Fig. 6L). Interestingly, we found that knockdown of BNIPL almost ablated corylin-activated GSDME (Fig. 6M), suggesting BNIPL also plays a critical role in corylin-triggered MM cell pyroptosis. Taken together, all the above results collectively showed that corylin promotes FOXO3 expression and activates GSDME, thereby exhibiting potent anti-MM activity by impairing mitochondrial function and inducing pyroptosis.

## Discussion

The above studies identify FOXO3 is a novel transcription factor of GSDME. FOXO3 promotes and activates GSDME therefore triggering GSDME-dependent MM cell pyroptosis by impairing mitochondrial function. Activation of the FOXO3/GSDME axis is a promising strategy to induce MM cell death through pyroptosis.

GSDME, one of the most important pyroptosis executors, is first identified as a deafness-associated gene because mutations in GSDME lead to familial autosomal dominant hearing loss 20. In cancer cells, GSDME has been reported as a tumor suppressor gene and its mutations lead to the loss of its anti-tumor activity 21. Moreover, GSDME expression is suppressed by hypermethylation in colorectal, gastric, and breast cancers 22. The present study finds that GSDME is strikingly downregulated in MM cells. However, this downregulation is not related to epigenetic modulations such as hypermethylation or acetylation, as no inhibitors of DNMT or HDACs are able to restore GSDME expression (Fig. 2A). Therefore, the molecular mechanisms underlying GSDME expression in MM cells might differ from those in other cancer cells. Transcription factors such as STAT3 and SP1 have been reported to upregulate GSDME expression, which contributes to GSDME-dependent pyroptosis 23],^24^. In the present study, we add that FOXO3 is also a transcription factor of GSDME.

The forkhead transcription factor FOXO3 displays a myriad of functions inlovled in cell apoptosis, DNA repair, and the oxidative stress response 25. In the present study, we found that FOXO3 induces MM cell pyroptosis in a GSDME-dependent manner. Specifically, FOXO3, as a transcription factor, binds to the promoter region of GSDME and upregulates its transcription. Similar to GSDME, FOXO3 is also downregulated in MM cells, and its low expression predicts poor prognosis of patients with MM. Although several reports have shown that GSDMD may mediate MM and AML cell pyroptosis 26, 27, FOXO3-induced MM cell pyroptosis is independent of GSDMD. This is because GSDMD is not activated by FOXO3 in MM cells, and FOXO3 fails to induce pyroptosis in MM cells that express GSDMD but not GSDME (Fig. 3) and this finding is consistent with previous reports 5,[8 in that GSDME is essential for cancer cell pyroptosis, particularly in chemically induced cell pyroptosis 5. Interestingly, in the present study, we also found that, in addition to inducing GSDME, FOXO3 also modulates the expression of Bcl-2-associated genes. Specifically, it downregulates Bcl-xL and upregulates Bcl-2-interacting proteins, including BNIPL, BNIP3 and BNIP3L, which have been previously reported to play roles in mitophagy 28. In the present study, we found that both BNIPL overexpression and Bcl-xL knockdown can trigger MM cell pyroptosis by activating BAX and impairing mitochondrial function. In line with previous studies 8, 29, the Bcl-2-associated proteins are important for GSDME-mediated cell pyroptosis. Therefore, in addition to their potential as biomarkers, FOXO3 and GSDME might serve as promising therapeutic target for MM, especially via the pyroptotic pathway.

Genetic studies have demonstrated that the restoration of GSDME significantly contributes to cancer treatment via pyroptosis and manipulation of GSDME activity has been identified as a novel route to treat cancer or other diseases 23, 30,31, but no chemicals have yet been identified that upregulate GSDME. In the present study, we found that corylin can markedly promote the expression of GSDME and FOXO3. Corylin is a natural product derived from the fruit of *Psoralea corylifolia L. (Fabaceae)*, a traditional Chinese herbal medicine. It displays various activities, including inducing osteoblastic differentiation 32, blocking UV-induced skin aging 33, and reducing obesity and insulin resistance 34. Corylin also shows great anti-cancer activities, such as suppressing lung cancer and colorectal tumor growth by inhibiting NF-κB 35 and STAT3 36, respectively. Therefore, corylin may display different activities via specific mechanisms in specific cell types. In this study, we further found that corylin shows potent anti-MM activity in both *in vitro* and* in vivo* models by inducing cell pyroptosis, specifically through upregulating the transcription of both FOXO3 and GSDME and impairing mitochondrial function. Given corylin has been successfully used in patients, the current study supports its promising application in cancer treatment such as myeloma.

## Conclusions

FOXO3 is a novel transcription factor of GSDME and both are downregulated in MM cells and predicts poor prognosis of MM patients. FOXO3 upregulates and activates GSDME to induce MM cell pyroptosis. Genetic or chemical induction of FOXO3 displays potent anti-MM activity by activating GSDME. The FOXO3/GSDME axis could be a promising strategy against MM via pyroptosis and impairing mitochondrial function.

## Supplementary Material

Supplementary figures and tables.
