# Supplementary material for: FOXO3 upregulates and activates GSDME to trigger myeloma cell pyroptosis
Source: Int J Biol Sci. 2026 Jan 15;22(4):1793–806. doi: 10.7150/ijbs.124782 (PMC12905583; doi:10.7150/ijbs.124782)
Supplement: Supplementary file 1 — Supplementary figures and tables. [file ijbsv22p1793s1.pdf]

For the submission:

FOXO3 upregulates and activates GSDME to trigger myeloma cell pyroptosis

Yaner Wang[1,2,#], Yali Wang[1,3,#], Yaoli Cui [1,#], Yuanming He[1], Ye Yang[4], Wen Zhou[5], Longlong Liu[1], Hua Wang[6], Mo Liu[7], Yongqiang Wei[8], Zhenqian Huang[1], Xiaolei Wei[8], Xinliang Mao[1,2,4*]

[1] Department of Hematology, The Key Laboratory of Advanced Interdisciplinary Studies, The First Affiliated Hospital of Guangzhou Medical University; Guangdong Provincial Key Laboratory of Protein Modification and Degradation, School of Basic Medical Sciences, Guangzhou Medical University, Guangzhou 511436, P. R. China

[2] Institute of Clinical Pharmacology, Science and Technology Innovation Center, Guangzhou University of Chinese Medicine, Guangzhou, 510405, China

[3] Department of Clinical Pharmacology, College of Pharmaceutical Sciences, Guangzhou Medical University, Guangzhou 511436, P. R. China

[4] School of Medicine & Holistic Integrative Medicine, Nanjing University of Traditional Medicine, Nanjing 210023, China

[5] Cancer Institute, Central China University, Changsha, 410078, P. R. China

[6] Department of Hematology, Southern Medical University, Guangzhou, 510515, P. R. China

[7] State Key Laboratory of Oncology in South China, Guangdong Provincial Clinical Research Center for Cancer, Sun Yat-sen University Cancer Center, Guangzhou, 510060, China

[8]Sino-French Hoffmann Institute, Guangzhou Medical University, Guangzhou 511436,

P. R. China

Supplemental Table S1. Specific primers for FOXO3 cloning

**Primers for FOXO3 cloning**	
Forward	5’-TCTAGAATGGACTACAAGGATGACGA-3’
Reverse	5’-GCGGCCGCTCAGCCTGGCACCCAGCTCT-3’
**Specific primers the construction of Tet-On FOXO3**	
Forward	5’-CCTACCCTCGTAAAGAATTCGCCACCATGGCAGAGGCACCGGCTTCCCCG-3’
Reverse	5’-AGGGGAGGTGGTCTGGATCCTCACTTGTCATCGTCATCCTTGTAATC-3’

Supplemental Table S2. Specific primers for RT-PCR for FOXO3, BAX, BCL2, BCL2A1, BCL2L1, BNIP3, BNIP3L, BNIPL, MCL1 and GAPDH

**Primers for FOXO3**	
Forward	5’- GCGTTGCGTGCCCTACTT -3’
Reverse	5’- CACTGACTGTGCTGGCGTTA-3’
**Primers for GSDME**	
Forward	5’- CCTCACCCTTGGCGATGT -3’
Reverse	5’- GGTCTGGATGCCCACGAT -3’
**Primers for GSDMD**	
Forward	5’- GACAACGTGTACGTGGTGACTG -3’
Reverse	5’- TGGAAGGTCCTCTGCTTCTTAT -3’
**Primers for BAX**	
Forward	5’- CCCGAGAGGTCTTTTTCCGAG-3’
Reverse	5’- CCAGCCCATGATGGTTCTGAT-3’
**Primers for BCL2**	
Forward	5’- GGTGGGGTCATGTGTGTGG-3’
Reverse	5’- CGGTTCAGGTACTCAGTCATCC-3’
**Primers for BCL2A1**	
Forward	5’-TACAGGCTGGCTCAGGACTAT -3’
Reverse	5’-CGCAACATTTTGTAGCACTCTG-3’
**Primers for BCL2L1**	
Forward	5’-GAGCTGGTGGTTGACTTTCTC-3’
Reverse	5’-TCCATCTCCGATTCAGTCCCT-3’

**Primers for BNIP3**	
Forward	5’-CAGGGCTCCTGGGTAGAACT -3’
Reverse	5’-CTACTCCGTCCAGACTCATGC-3’
**Primers for BNIP3L**	
Forward	5’-ATGTCGTCCCACCTAGTCGAG-3’
Reverse	5’-TGAGGATGGTACGTGTTCCAG-3’
**Primers for BNIPL**	
Forward	5’-GAGTCTGACTAAGGGGCCTG-3’
Reverse	5’-CTCCGAGTCTGAAGGTGTCT-3’
**Primers for MCL1**	
Forward	5’- GTAATAACACCAGTACGGACGG-3’
Reverse	5’- CCACAAACCCATCCTTGGAAG-3’
**Primers for GAPDH**	
Forward	5’- GGAAGATGGTGATGGGATT -3’
Reverse	5’- AACGGATTTGGTCGTATTG -3’

Supplemental Table S3: Primers for qRT-PCR of FOXO3, GSDME, and GAPDH

**Primers for FOXO3 (5’-3’)**	
Forward	5’-CGGACAAACGGCTCACTCT-3’
Reverse	5’-GGACCCGCATGAATCGACTAT-3’
**Primers for GSDME (5’-3’)**	
Forward	5-ACATGCAGGTCGAGGAGAAGT-3’
Reverse	5’-TCAATGACACCGTAGGCAATG-3’
**Primers for GAPDH (5’-3’)**	
Forward	5’-GGAGCGAGATCCCTCCAAAAT-3’
Reverse	5’-GGCTGTTGTCATACTTCTCATGG-3’

Supplemental Table S4:Primers for knockdown of FOXO3, BNIPL, and Bcl-xL

**Specific knockdown sequence for FOXO3**	
shFOXO3#1	5’-CCTACCCTCGTAAAGAATTCGCCACCATGGCAGAGGCA CCGGCTTCCCCG-3’
shFOXO3#2	5’-CGCTCGAAGTGGAGCTGGACCTTCAAGAGAGGTCCAG CTCCACTTCGAGCG

**Specific knockdownsequence for Bcl-xL**	
shBcl-xL #1<br>shBcl-xL #2	5'-GCTCACTCTTCAGTCGGAAAT-3'<br>5'-GTGGAACTCTATGGGAACAAT-3'
**Specific knockdownsequence for BNIPL**	
shBNIPL#1<br><br>shBNIPL#2	5'-GCTGGACAGTGGACATGAATTCTCGAGAATTCATGTCC ACTGTCCAGC-3'<br>5'-GCTGGATACGTCAGTGTTACCTTCAAGAGAGGTAACAC TGACGTATCCAGC-3'

Supplementary Table S5. The primers for the regulatory region of GSDME

**GSDME (1–2000): P0**	
Forward	5'-TGCAGGTGCCAGAACATTTCTCTATCGATAGGTACCCCCA AAGCACCCCAAGGCTGG-3'
Reverse	5'-AGGACACCTTCTTATTGCTTCTACTCATAACTAAAAGGTCT GCGGAGATTC-3'
**GSDME (-500~-1): P4**	
Forward	5'-TGCAGGTGCCAGAACATTTCTCTATCGATAGGTACCCCCA AA-3'
Reverse	5'-TGGTGGCTTTACCAACAGTACCGGAATGCCAAGCTTCATA AC-3'
**GSDME (-1000~-501): P3**	
Forward	5'-TGCAGGTGCCAGAACATTTCTCTATCGATAGGTACC-3'
Reverse	5'-TGGTGGCTTTACCAACAGTACCGGAATGCCAAGCTT-3'
**GSDME (-1501~–1001): P2**	
Forward	5'-TGCAGGTGCCAGAACATTTCTCTATCGATAGGTACC-3'
Reverse	5'-TGGTGGCTTTACCAACAGTACCGGAATGCCAAGCTT-3'
**GSDME (-2000~–1500): P1**	
Forward	5'-TGCAGGTGCCAGAACATTTCTCTATCGATAGGTACC-3'
Reverse	5'-TGGTGGCTTTACCAACAGTACCGGAATGCCAAGCTT-3'
**GSDME-△FRE1 (△1)**	
Forward	5'-CAATTTCTGCCTCCAAAGGAAGAATAAAAGGCAGAAATG AAATCCACAGG-3'
Reverse	5'-CTGTGGATTTCATTTCTGCCTTTTATTCTTCCTTTGGAGGC AGAAATTGG-3'
**GSDME-△FRE2 (△2)**	
Forward	5'-ATAGGTACCTATAGACTGTATTCTTATAAGAGAAAAGCAA ATGTTACGAAG-3'

Reverse	5’-TGGTGGCTTTACCAACAGTACCGGAATGCCAAGCTTGGGG GCTGCATGCAC-3’
**GSDME-△FRE1△FRE2 (GSDME-△1△2)**	
Forward	5’-GATAGGTACCTATAGACTGTATTCTTATAAGAGAAAAGCA AATGTTACGAAGAAAAAC-3’
Reverse	5’-GCCTTTTATTCTTCCTTTGGAGGCAGAAATTGGGCATAAGA CAATAC-3’

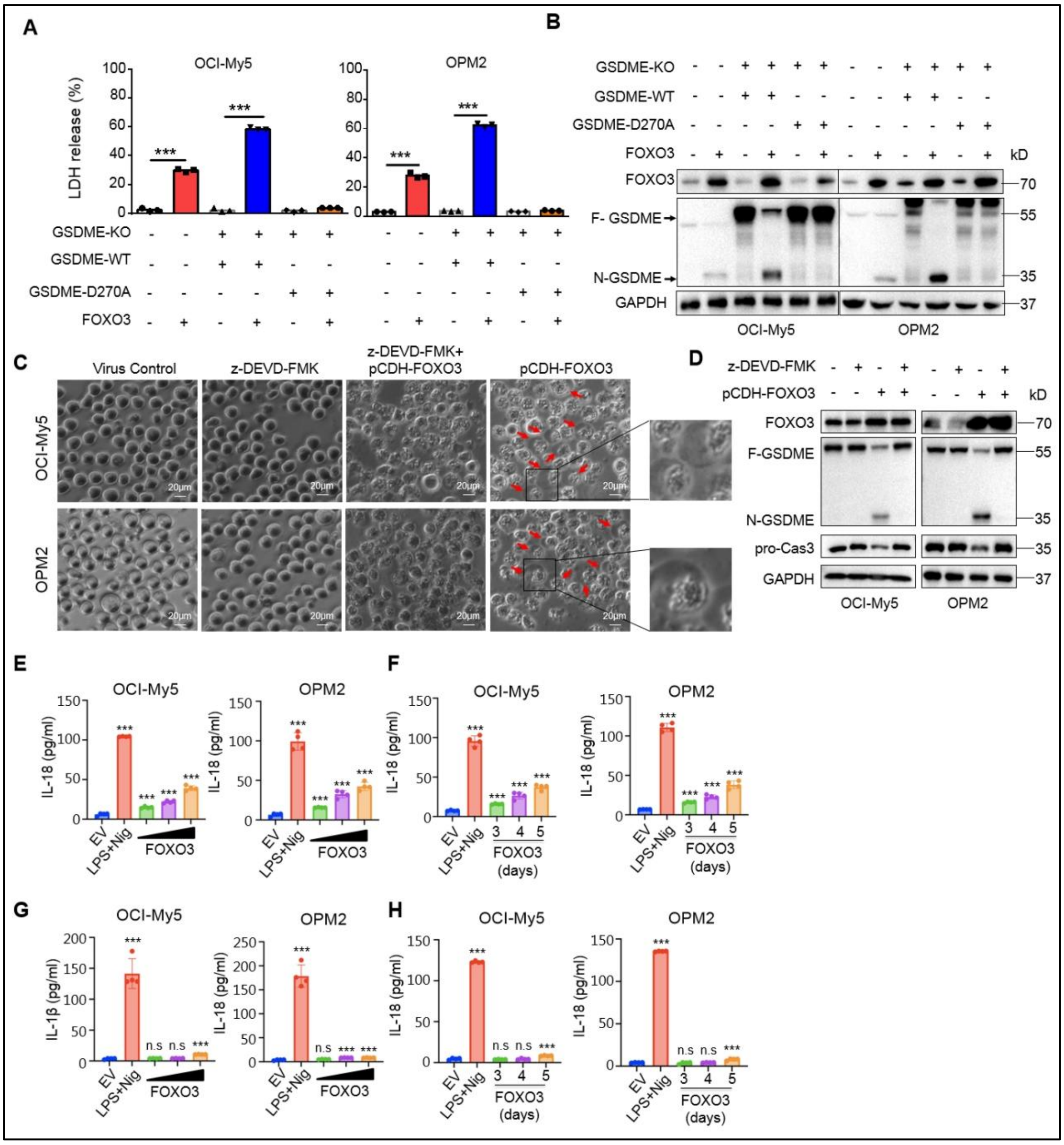

Suppl. Fig. S1. GSDME is required for FOXO3-induced myeloma cell pyroptosis. OCI-My5 and OPM2 were knocked out GSDME, followed by infection with FOXO3 along with wild-type or D270A mutant GSDME. A, LDH in culture media was measured. B, Cell

lysates were subjected to IB assays. C-D, OCI-My5 and OPM2 cells were infected with lentiviral FOXO3 for 72 h, followed by zDEVD-FMK treatment. Cell pyroptosis was subjected to Phase-contrast microscope analysis (C) and the cell lysates were subjected to WB for GSDME and Caspase-3 activation(D). E-F, MM cells were infected with lentiviral FOXO3 with increased titration (E) for 3 days or increased incubation days (F), followed by measurement of IL-18 by using an ELISA kit. LPS/Nigericin was used as a positive contol. G-H, MM cells were infected with lentiviral FOXO3 with increased viral particles of infection (G) for 3 days or increased incubation days (H), followed by measurement of IL-1β by using an ELISA kit. LPS/Nigericin was used as a positive contol.n.s., not significant. ***, $P < 0.001$.

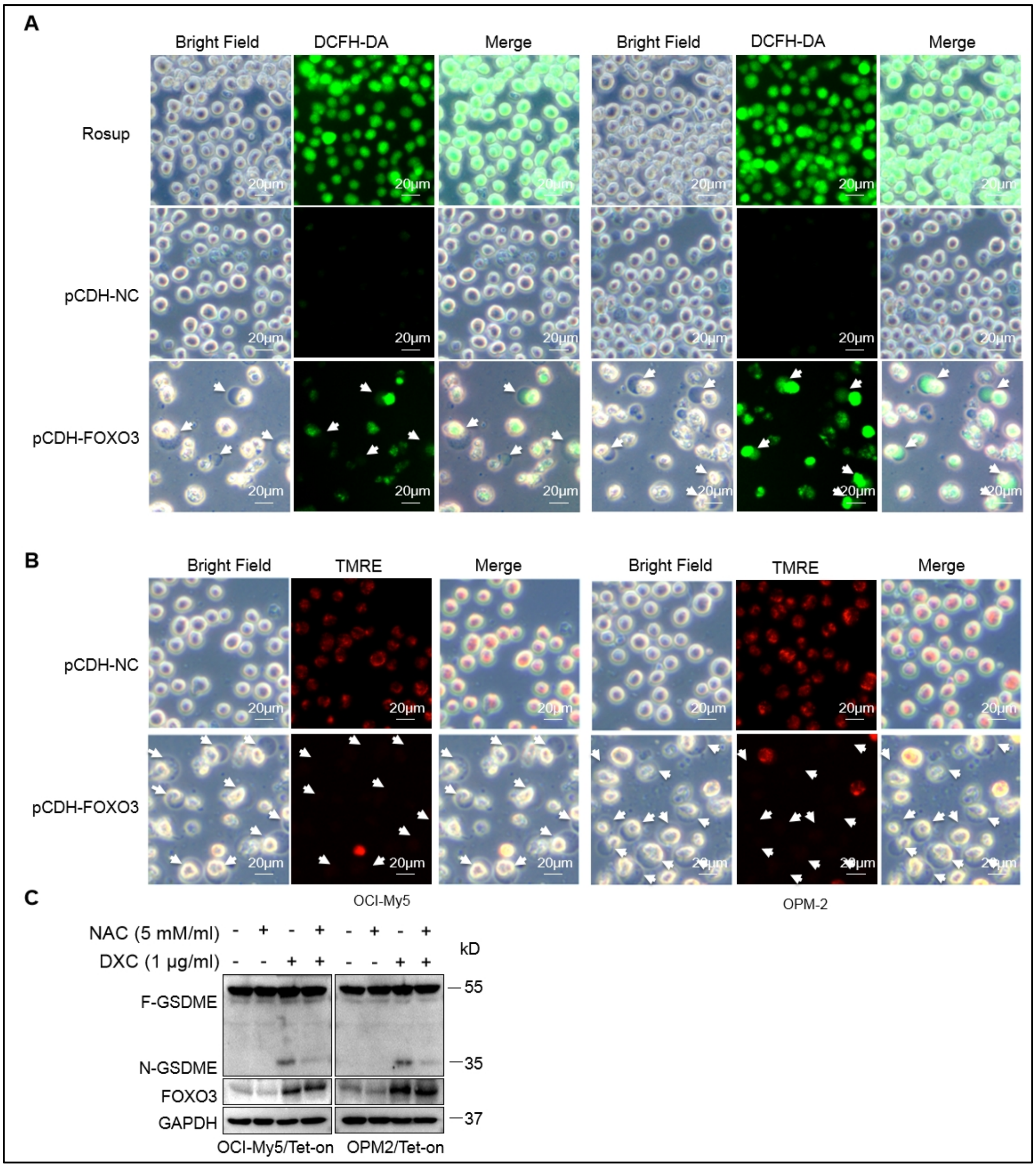

Suppl. Fig. S2. FOXO3 triggers ROS production and decreases mitochondrial membrane potential (MMP) in MM cells. A, MM cells were treated the positive control Rosup or infected with FOXO3 lentivirus. Cells were then stained with DCFH-DA to measure ROS production (A) or stained with TMRE to measure MMP (B). Arrows indicated pyroptotic cells.C, OCI-My5 and OPM2 stably infected with Tet-On/FOXO3 lentivirus were pre-treated with N-acetyl cysteine (NAC) for 5 h, followed by treatment with doxycycline (DXC) for 48 h. Cell lysates were then prepared for IB assays.

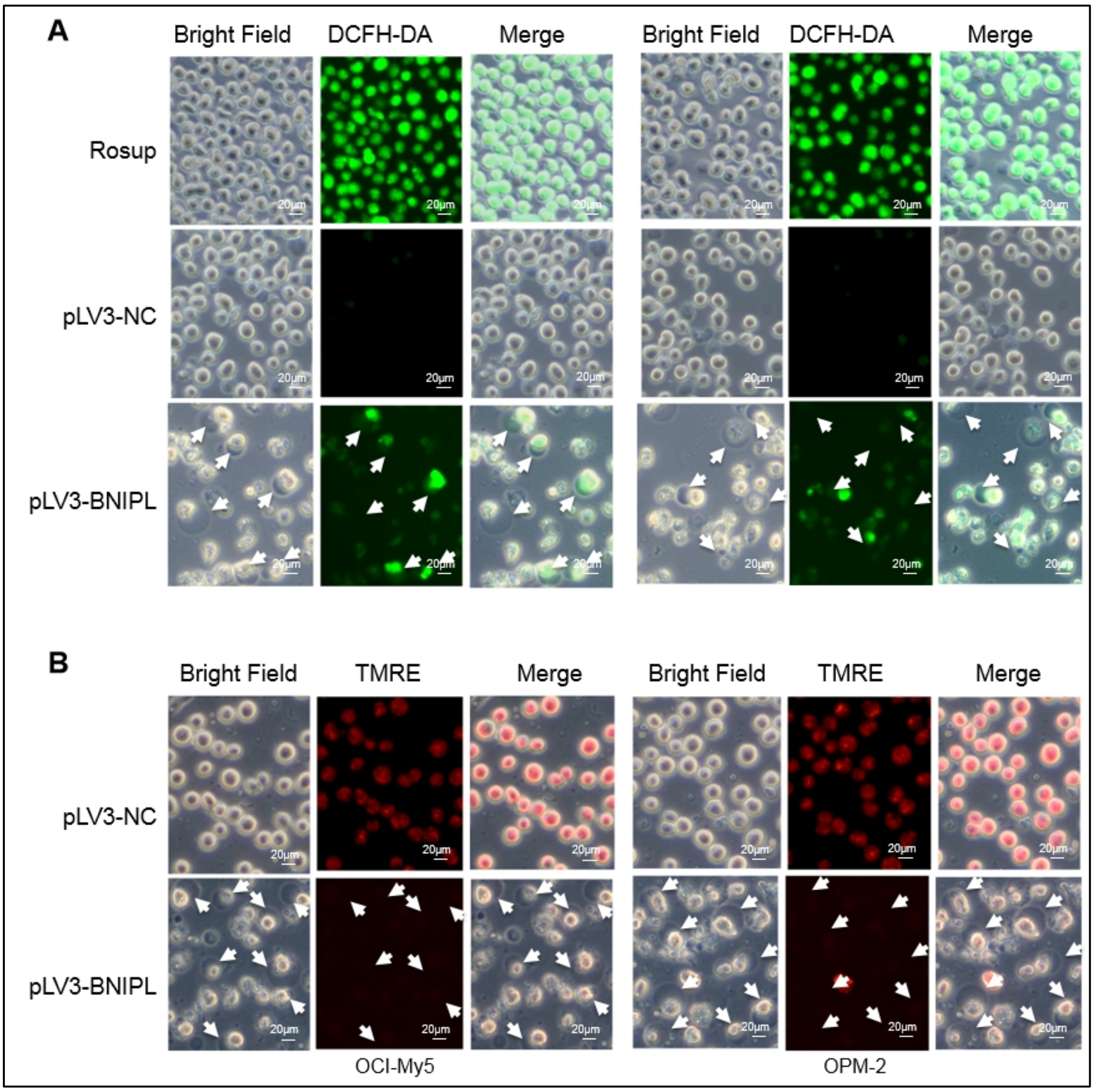

Suppl. Fig. S3. BNIPL overexpression elevated intracellular ROS levels and decreased MMP in MM cells. A, MM cells were treated the positive control Rosup or infected with BNIPL lentivirus. Cells were then stained with DCFH-DA to measure ROS production (A) or stained with TMRE to measure MMP (B). Arrows indicated pyroptotic cells.

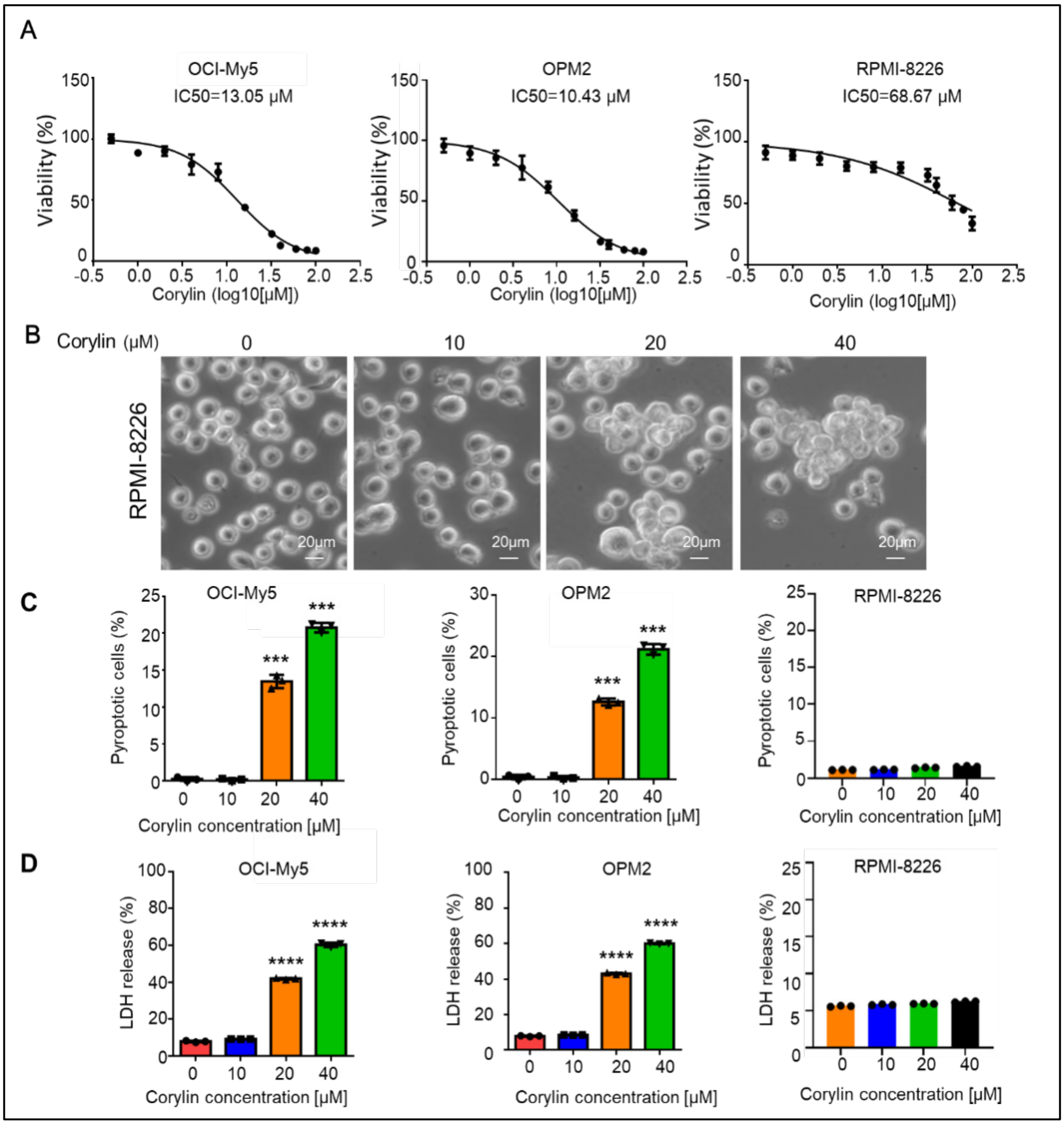

Suppl. Fig. S4. Corylin induces MM cell pyroptosis in a GSDME-dependent manner. A, OCI-My5, OPM2 and RPMI-8226 cells were incubated with increased corylin for 48 h followed MTT assay. IC50 was calculated. B, RPMI-8226 cells were treated with increased corylin for 24 h, followed by Phase-contrast microscopy analysis. C, Statistic analysis of pyroptotic cells from corylin-treated MM cells. D, LDH release into culture media after cells were treated with corylin was measured. ***, $P< 0.001$; ****, $P< 0.0001$.

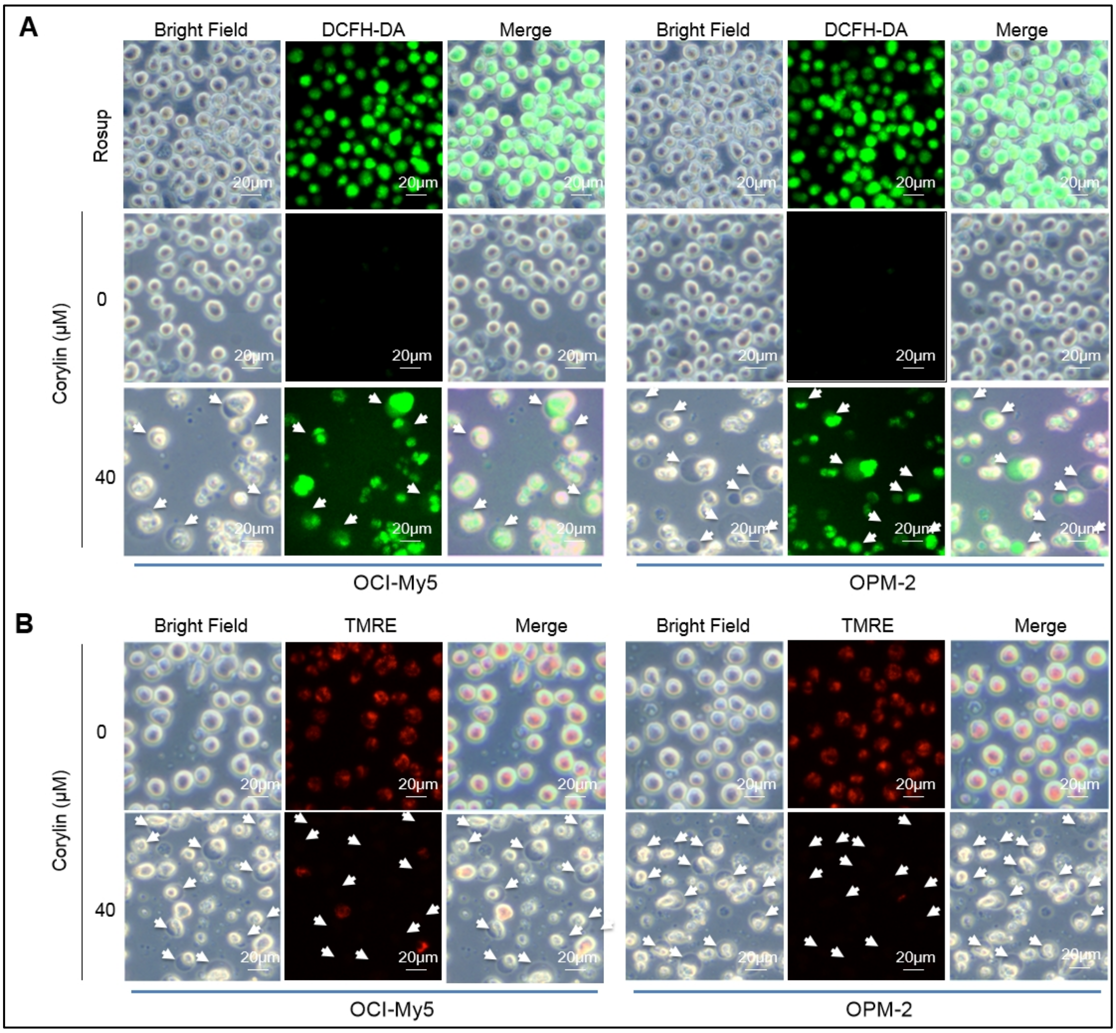

Suppl. Fig. S5. Corylin treatment elevates ROS levels and reduces MMP in MM cells. A-B, MM cells were treated the positive control Rosup or Corylin for 24 hrs. Cells were then stained with DCFH-DA to measure ROS production (A) or stained with TMRE to measure MMP (B). Arrows indicated pyroptotic cells.

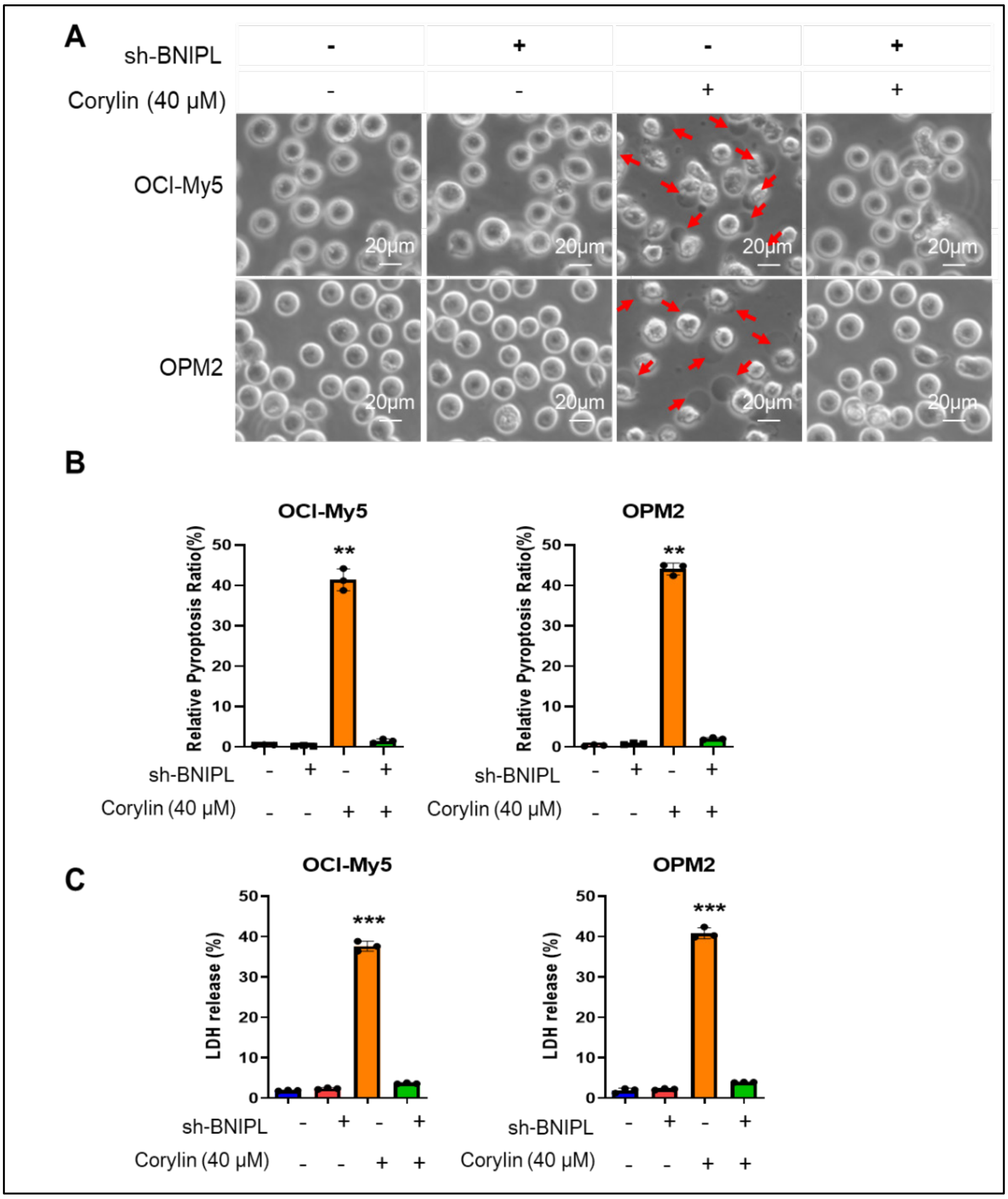

Suppl. Fig. S6. BNIPL is required for Corylin to induce MM cell pyroptosis. A-C, MM cells were knocked down BNIPL followed by incubation with increased corylin for 48 hrs. Cell pyroptosis was analyzed by Phase-contrast microscopy (A), pyroptosis ratio (B) and LDH measurement (C). ***, $P< 0.001$. Arrows indicated pyroptotic cells.
